# Reassessing the Role of DotF in the *Legionella pneumophila* Type IV Secretion System

**DOI:** 10.1371/journal.pone.0065529

**Published:** 2013-06-07

**Authors:** Molly C. Sutherland, Kelsey A. Binder, Phillip Y. Cualing, Joseph P. Vogel

**Affiliations:** 1 Department of Molecular Microbiology, Washington University School of Medicine, St. Louis, Missouri, United States of America; 2 Rocky Mountain National Institutes of Health Laboratories, Hamilton, Montana, United States of America; University of São Paulo, Brazil

## Abstract

*Legionella pneumophila*, the causative agent of a severe pneumonia termed Legionnaires’ Disease, survives and replicates within both protozoan hosts and human alveolar macrophages. Intracellular survival is dependent upon secretion of a plethora of protein effectors that function to form a replicative vacuole, evade the endocytic pathway and subvert host immune defenses. Export of these factors requires a type IV secretion system (T4SS) called Dot/Icm that is composed of twenty-seven proteins. This report focuses on the DotF protein, which was previously postulated to have several different functions, one of which centered on binding Dot/Icm substrates. In this report, we examined if DotF functions as the T4SS inner membrane receptor for Dot/Icm substrates. Although we were able to recapitulate the previously published bacterial two-hybrid interaction between DotF and several substrates, the interaction was not dependent on the Dot/Icm substrates’ signal sequences as predicted for a substrate:receptor interaction. In addition, binding did not require the cytoplasmic domain of DotF, which was anticipated to be involved in recognizing substrates in the cytoplasm. Finally, inactivation of *dotF* did not abolish intracellular growth of *L. pneumophila* or translocation of substrates, two phenotypes dependent on the T4SS receptor. These data strongly suggest that DotF does not act as the major receptor for Dot/Icm substrates and therefore likely performs an accessory function within the core-transmembrane subcomplex of the *L. pneumophila* Dot/Icm type IV secretion system.

## Introduction

*Legionella pneumophila* is a ubiquitous Gram-negative bacterium that survives and replicates within protozoa and human alveolar macrophages [1]–[5]. Inhalation of contaminated water droplets by humans can lead to a severe pneumonia called Legionnaires’ Disease [6], [7]. The ability of the bacterium to survive within host cells is dependent upon the secretion of approximately two hundred and seventy-five protein substrates into the host cell [8]–[12]. This vast array of substrates has a wide variety of functions, including creation of a replicative niche for the bacterium, prevention of phagosome-lysosome fusion and evasion of the host immune system (reviewed in [6], [13]–[17]).

**Figure 1:**
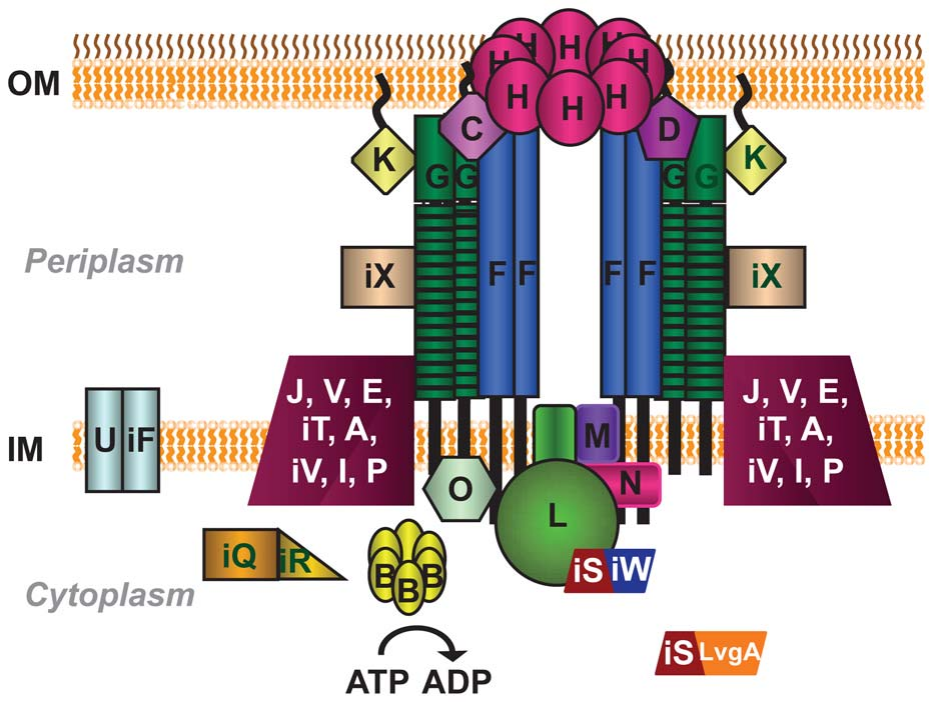
Schematic of the *L.* *pneumophila* type IV secretion system (T4SS). The 27 proteins of the T4SS are shown at their predicted or experimentally determined subcellular locations in the outer membrane (OM), periplasm, inner membrane (IM) and cytoplasm. Dot proteins are labeled with the last letter of their name. Icm proteins are designated in the same manner, but are prefaced with an ‘i’.

While a significant amount of research has focused on discovering functions for the translocated substrates, less effort has been dedicated to understanding their mechanism of secretion. It is known that export of substrates into host cells requires a type IV secretion system (T4SS), designated Dot/Icm for defect in organelle trafficking or intracellular multiplication defect [18], [19]. The Dot/Icm T4SS is composed of twenty-seven proteins and the subcellular localization of twenty-two of these proteins has been experimentally established (summarized in Fig. 1) [20]. A significant number of the Dot/Icm proteins are organized into two major protein subcomplexes: the type IV coupling protein (T4CP) subcomplex and the core transmembrane subcomplex [20], [21].

The T4CP subcomplex consists of DotL(IcmO), DotM(IcmP), DotN(IcmJ), and the heterodimer pair IcmS/IcmW (referred to as IcmSW for the remainder of the text) [21]. In type IV secretion systems, the coupling protein family has been shown to bind to substrates in the cytoplasm and then ‘couple’ them to the T4SS apparatus. In addition, T4CPs contain a Walker A Box motif for ATP hydrolysis and therefore are thought to provide energy to pump substrates across the bacterial membrane(s) and out of the cell (reviewed in [22]). Based on a similar membrane topology, the presence of a Walker A Box motif, and limited homology to known T4CPs, DotL was proposed to be the Dot/Icm T4CP and function as the receptor to recognize substrates expressed within the bacterial cytoplasm [23]. Recently it was shown that IcmSW, type IV adaptor proteins that are known to interact with a subset of Dot/Icm substrates and be required for their export [24]–[28], also directly bind to DotL [29]. Interestingly, the interaction between IcmSW and DotL is required for the specific translocation of IcmSW-dependent substrates [29], supporting the model that DotL functions as the T4CP for the Dot/Icm type IV secretion system.

In contrast to the inner membrane T4CP subcomplex, the core transmembrane subcomplex bridges both the inner and outer membranes of *L. pneumophila* [20]. It is composed of DotC, DotD, DotF(IcmG), DotG(IcmE) and DotH(IcmK). DotH was proposed to form the outer membrane pore of the T4SS, whereas DotC and DotD are lipoproteins that assist in the proper localization of DotH. Although DotF and DotG were shown to be inner-membrane proteins, they fractionate with the outer membrane due to their interactions with DotC, DotD, and DotH [20]. Based on its homology to the *Agrobacterium tumefaciens* protein VirB10 and relatedness to the *Escherichia coli* protein TonB, both of which transduce energy from the inner-membrane to the outer-membrane [30], [31], DotG likely transfers energy from the inner membrane to the putative DotH pore [20]. DotF was shown to interact with DotG and thus was postulated to play a critical role in the subcomplex by regulating DotG’s energy transducing activity [20].

In addition to its role in the core transmembrane subcomplex, DotF has been described as potentially having two alternate functions. First, it was proposed to contain a SNARE-like motif at amino acids 146–210 [32] that was able to inhibit membrane fusion when included in an *in* *vitro* assay [33]. However, no evidence exists that DotF is secreted and this concept is inconsistent with experimental evidence demonstrating that DotF is localized to the bacterial inner-membrane [20]. Second, an interaction between DotF and Dot/Icm substrates was observed in a bacterial two-hybrid screen [34], suggesting that DotF directly binds substrates during the export process. Based on the localization of DotF and substrates, the most likely explanation for this interaction would be if DotF functions as the cytoplasmic receptor for the Dot/Icm type IV secretion system. To test this model, we further characterized the interaction between DotF and substrates.

## Results

DotF is a protein localized to the bacterial inner membrane [20]. It consists of 269 amino acids and contains three domains: an n-terminal cytoplasmic domain of 50 amino acids, ∼20 amino acids that span the cytoplasmic membrane, and a c-terminal ∼200 amino acid periplasmic domain. In a previous study [34], a DotF fragment was isolated from a library of *L. pneumophila* genomic clones due to its ability to interact with the Dot/Icm substrate RalF. The DotF fragment consisted of amino acids 29–123 (referred to as DotF(29–123) for the remainder of the text) and thus contained a portion of the cytoplasmic domain, the transmembrane domain, and approximately one quarter of the periplasmic domain. This DotF fragment was subsequently used to rescreen the *L. pneumophila* library resulting in the identification of eight proteins that were designated “Sids” for substrates of *icm/dot* system [34].

**Figure 2:**
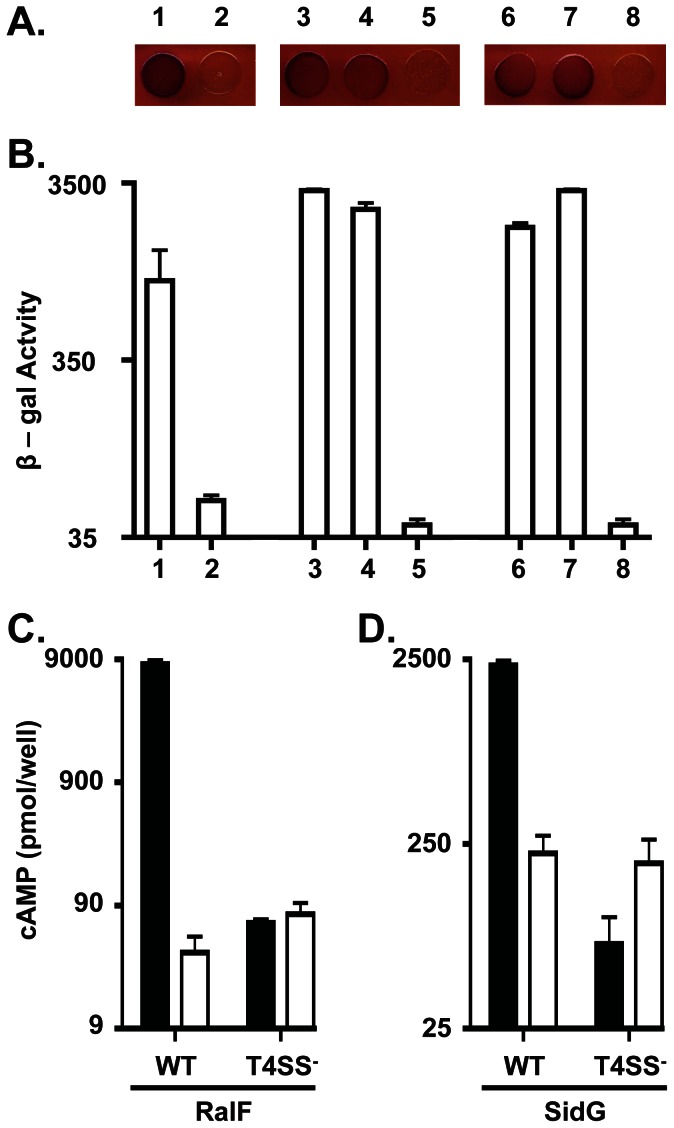
Interaction between DotF and substrates is not mediated by the secretion signal sequence. (A) Visualization of the two-hybrid interaction on MacConkey indicator media. Shown is a portion of a plate containing a red positive control consisting of two leucine zippers fused to the T18 and T25 domains of CyaA (1) and a white negative control consisting of the T18 and T25 empty vector controls (2). The T18:DotF(29–123) fragment was assayed for interaction with T25:RalF and T25:SidG containing their signal sequences (3–4) versus fusions lacking their signal sequences (6–7) and the T25 vector control (5 and 8). (B) Quantification of the interactions in (A) by measurement of β-galactosidase activity. (C,D) The secretion signal sequence is required for translocation. CyaA:RalF (black bars), CyaA:RalF(ΔSS) (white bars), CyaA:SidG (black bars), CyaA:SidG(ΔSS) (white bars) were assayed for secretion in a wild type strain (WT) or a *dotA* mutant strain that is defective for secretion (T4SS^−^). Secretion was monitored by an ELISA assay that detects calmodulin-induced cAMP production. Assays were performed in triplicate and error bars represent the standard deviation from the mean.

To further probe DotF’s interaction with substrates and to ascertain if DotF serves as the receptor for substrates, we re-examined potential interactions between the DotF(29–123) fragment and fifteen Dot/Icm substrates using the same bacterial two-hybrid assay [35]. This assay is based on the reconstitution of *Bordetella pertussis* adenylate cyclase (CyaA) activity when fusions to two CyaA domains (T18 and T25) are brought into proximity due to a protein:protein interaction. A positive interaction results in the formation of a red pigment on MacConkey agar supplemented with maltose due to fermentation of the sugar (Fig. 2A) [35]. Alternatively, productive interactions can be quantified through measurement of β-galactosidase activity, which is also dependent on CyaA function (Fig. 2B) [35]. In our hands, an interaction could be detected between six Dot/Icm substrates and DotF(29–123), although nine additional substrates failed to generate a positive signal (Fig. 2A–B and Table S1). The interactions appeared to be genuine as they were observed when the Ladant T18 and T25 fragments were swapped between the bait and prey, i.e. the interactions were reciprocal (data not shown). Binding between DotF and RalF, SidF and SidG had been observed before [34], whereas we were able to identify three additional DotF:substrate interactions involving SidJ, SdeA, and LnaB (Fig. 2A–B and Table S1). The Ladant T18 vector did not interact with the T25:DotF(29–123) fusion, indicating that the interactions were specific (Fig. 2A–B). Similar to the previous report [34], we discovered that the substrates were able to interact with fusions to full-length DotF(1–269), although at a lower level (data not shown).

Upon confirming the original observation that an interaction could be detected between DotF and Dot/Icm substrates, we explored in more detail the possibility that DotF functions as the cytoplasmic receptor for substrates. If DotF performs this role, then it should fulfill a set of parameters that include the following criteria. First, a receptor should recognize and engage substrates via their signal sequence. Second, a receptor should be absolutely required for the intracellular survival of *L. pneumophila*. Lastly, strains lacking the receptor should be unable to secrete Dot/Icm substrates.

Although the signal sequence for Dot/Icm substrates is not well characterized, it is believed to be located at the c-terminus of these proteins [36]. For example, RalF’s signal sequence was mapped to its last 20 amino acids [37] whereas SidG’s signal sequence was mapped to its c-terminal 35 amino acids [25]. Therefore, we constructed new Ladant fusions containing RalF and SidG that lack their signal sequences (designated as RalF(ΔSS) and SidG(ΔSS), respectively). Surprisingly, RalF lacking its signal sequence and SidG lacking its signal sequence still interacted with the DotF(29–123) fragment (Fig. 2A–B). The interactions were similar to that observed using substrates containing their c-termini (Fig. 2A–B), indicating that the observed DotF:substrate interaction was not mediated via their signal sequence. To confirm that we had successfully removed the signal sequences, the deletions were fused to full length CyaA and assayed for secretion [24], [29]. As previously shown [25], [37], both RalF(ΔSS) and SidG(ΔSS) were not secreted as they had the same level of signal as observed for the wild type proteins in a T4SS-deficient strain (Fig. 2C–D), thus verifying that we had removed the signal sequence from each protein. Finally, consistent with the observation that DotF is able to recognize RalF in the absence of its signal sequence, a Ladant fusion containing only the RalF signal sequence was not able to interact with DotF (data not shown).

**Figure 3:**
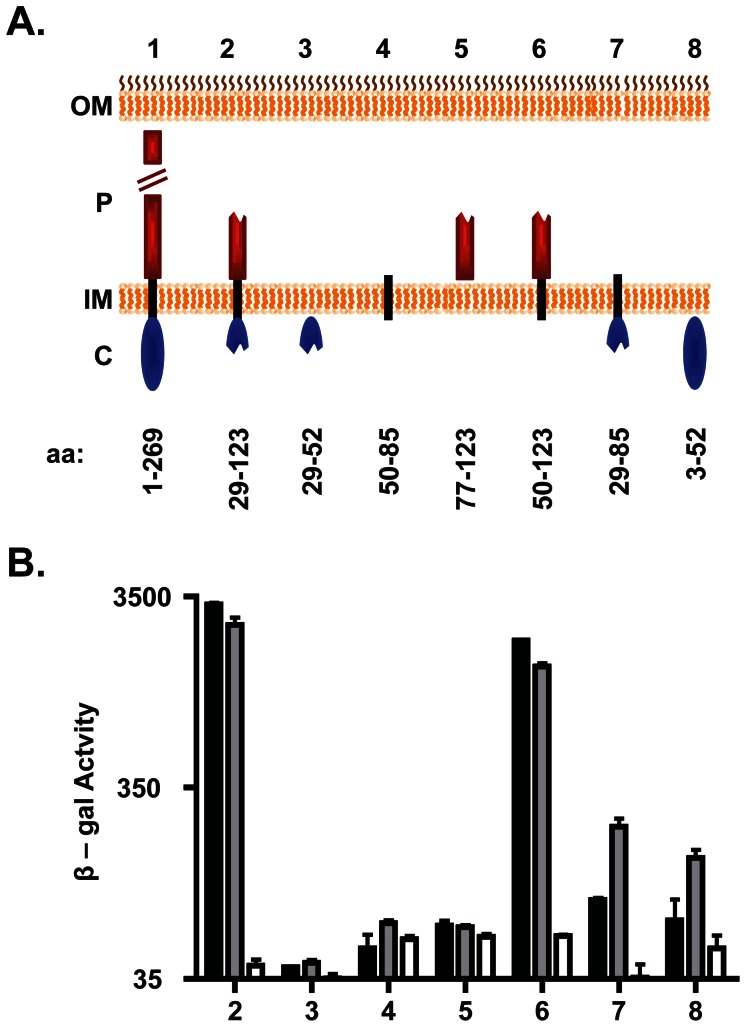
DotF interacts with substrates via its periplasmic and transmembrane domains. (A) Schematic of the DotF fragments used to determine the DotF-substrate interaction domain. The outer membrane (OM), periplasm (P), inner membrane (IM) and cytoplasm (C) are shown. DotF was divided into three domains: cytoplasmic (blue ovals), transmembrane (black rectangle), and periplasmic (red rectangles). Full-length DotF (1) and the DotF fragments (2–8) are shown and their amino acid range is provided beneath the schematic. (B) The DotF fragments (2–8) were tested for interaction with RalF (black bars), SidG (gray bars) or the vector (white bars) by measurement of β-galactosidase activity. Assays were performed in triplicate and error bars represent the standard deviation from the mean.

These unanticipated results prompted us to examine the bacterial two-hybrid interaction between DotF and substrates in more depth, specifically by determining which domain of the DotF(29–123) fragment was responsible for binding substrates. Based on the cytoplasmic expression of T4SS substrates, we postulated that substrates would interact with the n-terminal domain of DotF that is positioned in the cytoplasm [20]. Surprisingly, neither RalF nor SidG was able to interact with a Ladant fusion to DotF(29–52), which contained the cytoplasmic portion of the original DotF fragment that we anticipated would interact with substrates (Fig. 3B). In addition, both substrates did not interact with the DotF fragments containing just the transmembrane domain or the relevant portion of the periplasmic domain (Fig. 3B). Consequently, we examined combinations of domains in case the interaction required more than one domain or the presence of additional domains were needed for proper membrane insertion of the bait. Unexpectedly, the DotF fragment containing the transmembrane and periplasmic domains (DotF(50–123)) exhibited nearly the same level of signal with both substrates as the original DotF(29–123) fragment (Fig. 3B). This result was not foreseen since DotF(50–123) lacks the cytoplasmic domain predicted to be required for interaction with substrates in the cytoplasm.

The above data suggested that the two-hybrid signal generated is surprisingly due to an interaction solely between substrates and the transmembrane and/or periplasmic domains of DotF. We did observe a modest signal between SidG and a DotF fragment consisting of the cytoplasmic and transmembrane domains (DotF(29–85)), although it was significantly less than observed with the original DotF(29–123) fragment (Fig. 3B). In contrast, limited signal was observed between RalF and DotF(29–85). Finally, we tested if a fusion containing the complete cytoplasmic domain of DotF was capable of interacting with substrates in this bacterial two-hybrid assay. Once again we noted a minimal interaction with SidG, whereas RalF generated no signal (Fig. 3B). All of the DotF fragments produced equivalent amounts of protein (Fig. S1), indicating that the inability to interact with substrates by some fragments was not due to decreased protein levels. In summary, it appears that the DotF cytoplasmic domain does not play a major role in recognizing substrates in the two-hybrid assay as projected for a membrane receptor for soluble substrates expressed in the cytoplasm.

**Figure 4:**
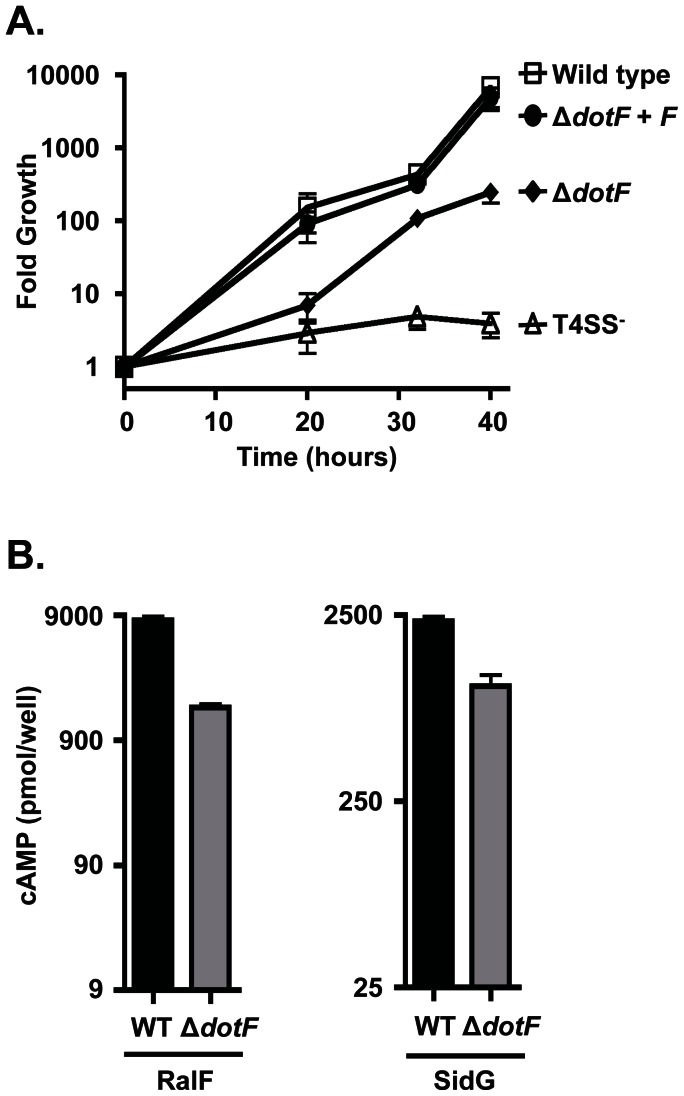
Strains lacking DotF retain the ability to partially grow intracellularly and secrete Dot/Icm substrates. (A) Δ*dotF* exhibits a partial growth phenotype in *A. castellanii*. A wild type strain (open squares), a secretion incompetent strain (open triangles), a strain lacking *dotF* (filled diamonds) and a Δ*dotF* complemented strain (filled circles) were used to infect *A. castellanii*. Intracellular growth was monitored by CFU determination at the time of infection and 20, 32 and 42 hours post infection. Infections were performed in triplicate and fold growth was determined by dividing the CFUs at a given time point by the CFUs at time zero. Error bars represent the standard deviation from the mean. (B) A Δ*dotF* mutant strain is able to secrete Dot/Icm substrates. Full-length RalF and SidG fusions to CyaA were assayed for secretion in a wild type strain (WT, black bars) or a strain lacking *dotF* (Δ*dotF*, gray bars). Data was generated in the same secretion experiment show in Figure 2.

Based on these experiments, we concluded that the observed interaction between DotF and substrates was not mediated by the substrates’ signal sequence, nor by DotF’s cytoplasmic domain, thus calling into question the hypothesis that DotF serves as a substrate receptor for the Dot/Icm T4SS. Nevertheless, we went on to further test this hypothesis by assessing the effect of a *dotF* deletion on the intracellular growth and secretion by *L. pneumophila*. To perform this experiment, a strain containing a large internal in-frame deletion of *dotF* was constructed [20]. The ability of the Δ*dotF* mutant to replicate within the host cell *Acanthamoeba castellanii* was compared to a wild type strain and a strain containing a mutation in *dotA* (Lp03) that inactivates the Dot/Icm T4SS. Consistent with previous publications [19], [29], the wild-type strain was able to increase approximately 5000 fold over 40 hours (Fig. 4A, open squares) while the *dotA* mutant was severely attenuated for intracellular replication (Fig. 4A, open triangles). In contrast, the Δ*dotF* strain exhibited a significant amount of growth compared with the *dotA* mutant (filled diamonds), providing evidence that *dotF* was not essential for intracellular growth. This strain could be fully complemented when *dotF* was expressed from a plasmid (filled circles), illustrating that our deletion did not have polar effects on the downstream genes in the operon. Thus, *dotF* is not absolutely required for intracellular growth as predicted if it was the substrate receptor.

Lastly, we examined the requirement of *dotF* for optimal export of substrates into a host cell. Translocation of CyaA fusions to RalF and SidG were assayed again using an adenylate cyclase reporter assay [24], [29], [38]. CyaA fusions to RalF and SidG were robustly secreted from a wild type strain (Fig. 4B, black bars). Notably, the Δ*dotF* mutant (gray bars) retained the ability to secrete substrates, albeit at a slightly lower activity than the wild type strain. As there is a significant level of export in the Δ*dotF* mutant, it is improbable that DotF functions as the major substrate receptor for the Dot/Icm T4SS.

## Discussion

In this study, we examined the theory that DotF serves as the receptor that binds and targets cytoplasmically expressed substrates to the Dot/Icm T4SS. We systematically tested a number of criteria predicted for a receptor including its ability to interact with a substrate signal sequence, the topological requirement for a domain to bind substrates in the cytoplasm, and predicted phenotypes involving intracellular growth and secretion dependency.

We began by testing if DotF was able to interact with a random collection of Dot/Icm substrates, a trait consistent with the notion that it serves as the substrate receptor. We were able to confirm an interaction between the DotF(29–123) fragment and RalF and were able to detect an interaction with five other substrates (Table S1). However, DotF failed to bind to nine additional substrates, thus calling into question the prevalence of this interaction. It is worth noting that the original two-hybrid report identified 68 putative DotF-interacting proteins after screening 150,000 clones, although only 8 of these were confirmed to be Dot/Icm substrates [34]. Considering that *L. pneumophila* expresses a large number of Dot/Icm substrates, currently estimated to be about 10% of the proteome, it is not clear if the previous screen possessed a significant level of selective power.

Nevertheless, we carefully re-examined the relationship between DotF and substrates. Although we could detect an interaction between DotF and some substrates, the interaction was not dependent on the substrates’ signal sequences (Fig. 2). Moreover, the substrates did not interact with the cytoplasmic portion of DotF (Fig. 3), the domain predicted to bind substrates expressed in the cytoplasm of bacteria. Taken together, these data strongly suggest that DotF is not the cytoplasmic receptor for Dot/Icm substrates. Alternatively, it is possible that the two-hybrid signal might be due to an interaction between a second domain of substrates, distinct from their c-terminal signal sequence, which interacts with a periplasmic domain of DotF during the export process. However, the bacterial two-hybrid assay we used requires both adenylate cyclase fragments to interact in the cytoplasm, thus topologically precluding an interaction between a cytoplasmic substrate and a periplasmic domain of an inner membrane protein. Therefore, it is difficult to reconcile how such an interaction could be detected by this assay, thus calling into question the biological relevance of the original two-hybrid interaction.

To further disprove the hypothesis that DotF functions as the substrate receptor, we re-examined the intracellular growth requirement for DotF. Previously, DotF was shown to be only partially required for intracellular growth of *L. pneumophila* within HL-60 cells and guinea pig alveolar macrophages [39]–[41]. In contrast, DotF was reported to be completely required for growth within *A. castellanii* [19]. However, the insertional *dotF* mutant that was used in that study could not be fully complemented by exogenous expression of *dotF* [19], suggesting the strain contained an unlinked mutation that exacerbated the intracellular growth defect. Consistent with this possibility, our *dotF* deletion was only partially attenuated for growth in *A. castellanii* and could be fully complemented by expression of wild-type *dotF*, indicating that *L. pneumophila* does not absolutely require *dotF* for replication within a host. Furthermore, a separate research group showed that their *dotF* mutant was only partially defective for growth in the amoebae plate test (APT) [42]. Likewise, our Δ*dotF* strain retained the ability to secrete both RalF and SidG. Taken together, these results indicate that DotF does not function as a key component of the Dot/Icm T4SS, a role that would be expected if DotF acts as the receptor for substrate recognition in the cytoplasm.

Based on these results, we propose that DotL more likely serves as the major receptor for substrates than DotF. DotL displays homology to T4CPs [23], which have been decisively shown to function as receptors in many T4SSs (reviewed in [22]). Moreover, *dotL* is absolutely required for growth inside host cells [43] and has been shown to interact with the type IV adaptors IcmSW to regulate secretion of IcmSW-dependent substrates [21], [29], a trait consistent with its role as a substrate receptor. Proof that DotL is the major receptor for Dot/Icm substrates has not been obtained since a direct interaction between DotL and substrates has proven to be experimentally difficult to confirm. This failure is likely due to the vast numbers of substrates expressed in *L. pneumophila*, thus preventing concurrent stable interactions between the numerous substrates and DotL [8]–[12]. As the receptor for the Dot substrates must perform an essential role in regulating the orderly export of the large number of T4SS substrates expressed by this pathogen, further work in this area is warranted.

## Materials and Methods

### Bacterial Strains, Plasmids and Media

Strains and plasmids used in this study are provided in Table S2. Plasmid construction is described in Table S3. All *Legionella pneumophila* strains are designated with a JV number and have been derived from the wild type derivative, *L. pneumophila* Philadelphia Lp02 (*hsdR rpsL thyA*), of the clinical isolate *L. pneumophila* Philadelphia-1 [44]. *L. pneumophila* strains were grown on solid media consisting of charcoal yeast agar (CYE) or in liquid yeast extract broth (AYE), both buffered with *N*-(2-acetamido)-2-aminoethanesulfonic acid (ACES) [45]. Antibiotics and thymidine were added as needed. Protein expression of Cya fusions (translocation assays) and *dotF* (rescue experiment) in *L. pneumophila* was induced by the addition of 100 µM IPTG prior to infection for several hours or overnight, respectively.

### Bacterial Two-hybrid

Bacterial two hybrids were performed as previously described [20], [34], [35]. The original DotF fragment identified by Luo and Isberg was isolated from a Sau3AI-digested *L. pneumophila* genomic library and the authors designated this fragment as beginning with amino acid 28 of DotF [34]. However, as *dotF* contains a Sau3AI site at bp 85–88, the fusion would begin with Asp29, not Ser28. As a result, we constructed our initial DotF fragment to start at Asp29 and refer to it as DotF(29–123). Since Luo and Isberg demonstrated that a c-terminal fragment of RalF exhibited a better interaction with DotF than full-length RalF [34], we used a c-terminal fragment of RalF consisting of amino acids 190–374 in the bacterial two hybrid assays.

To assess protein-protein interaction, the two proteins of interest were fused to either the n-terminal fragment (T18) or the c-terminal fragment (T25) of *B. pertussis* adenylate cyclase (CyaA) and were then transformed into *E. coli* BTH101 (F^-^, *cya-99*, *araD139*, *galE15*, *glaK16*, *rpsL1*, *hasdR2*, *mcrA1*, *mcrB1*). Transformants were selected on Luria Broth media containing the appropriate antibiotics. Subsequently, the transformants were spotted on MacConkey media supplemented with 1.0% maltose. Reconstitution of CyaA allows for fermentation of maltose, resulting in a red pigment. Strains were constructed independently for each experiment.

The protein levels of the T18:DotF fragments were assessed by induction in *E. coli* BL21. The strains were induced with IPTG and grown to stationary phase, samples were collected and protein levels were ascertained by western blot with a CyaA specific antibody (sc-13582, Santa Cruz Biotechnology).

### Quantitative Assay for Protein-protein Interaction (β-galactosidase Activity)

*E. coli* BTH101 strains expressing a T18 and T25 fusion plasmid were grown overnight at room temperature under inducing conditions. Stationary phase cultures were pelleted and resuspended in 1 ml working buffer (61 mM Na2HPO4, 39 mM NaH2PO4, 10 mM KCl, 10 mM MgSO4-7H2O, 0.35% 2-Mercaptoethanol (BME)), the optical density (OD) 600 was measured. Cells were lysed, incubated with 200 µl ortho-Nitropheny-β-galactoside (ONPG) and the reaction was stopped once it turned yellow with 1 M sodium carbonate. The lysate was cleared, the OD420 was measured and the Miller Units were calculated with the following formula ((OD420×1000)/(time × volume of cells × OD600).

### Intracellular Growth in *A. castellanii*

Intracellular growth assays were performed as previously described [19], [46]. Briefly, *L. pneumophila* liquid cultures were grown to the point of optimal virulence (OD600 ∼3.3) and were used to infect *A. castellanii* cells. Amoeba were lysed at 0, 20, 32 and 42 hours with 0.5% saponin in PBS and dilutions were plated to obtain colony forming units (CFU). Fold growth was determined by dividing the number of CFUs at the designated time point by number of CFUs at time zero.

### Adenylate Cyclase Reporter Assays for Substrate Secretion

U937 cells [47] were prepared as previously described [48] and adenylate cyclase reporter assays were performed [24], [29], [38]. Extracted cAMP was desiccated and quantified using a competitive ELISA (Cyclic AMP EIA Kit, Cayman Chemical).

## Supporting Information

Figure S1 The T18:DotF two-hybrid constructs produce equivalent amounts of protein. The T18:DotF protein levels were assessed by western blot with a CyaA-specific antibody. The T18 CyaA fragment expressed from the vector is indicated by an arrow. The T18:DotF fragments are designated by a bracket and their amino acid range is indicated above the western blot.
(TIF)

Table S1 Dot/Icm T4SS substrates tested for interaction with DotF(29–123).
(PDF)

Table S2 Strains, plasmids and primers employed in this study.
(PDF)

Table S3 Construction of plasmids employed in this study.
(PDF)
