# Supplementary figures and images for: Reassessing the Role of DotF in the Legionella pneumophila Type IV Secretion System
Source: PLoS One. 2013 Jun 7;8(6):e65529. doi: 10.1371/journal.pone.0065529 (PMC3676331; doi:10.1371/journal.pone.0065529)

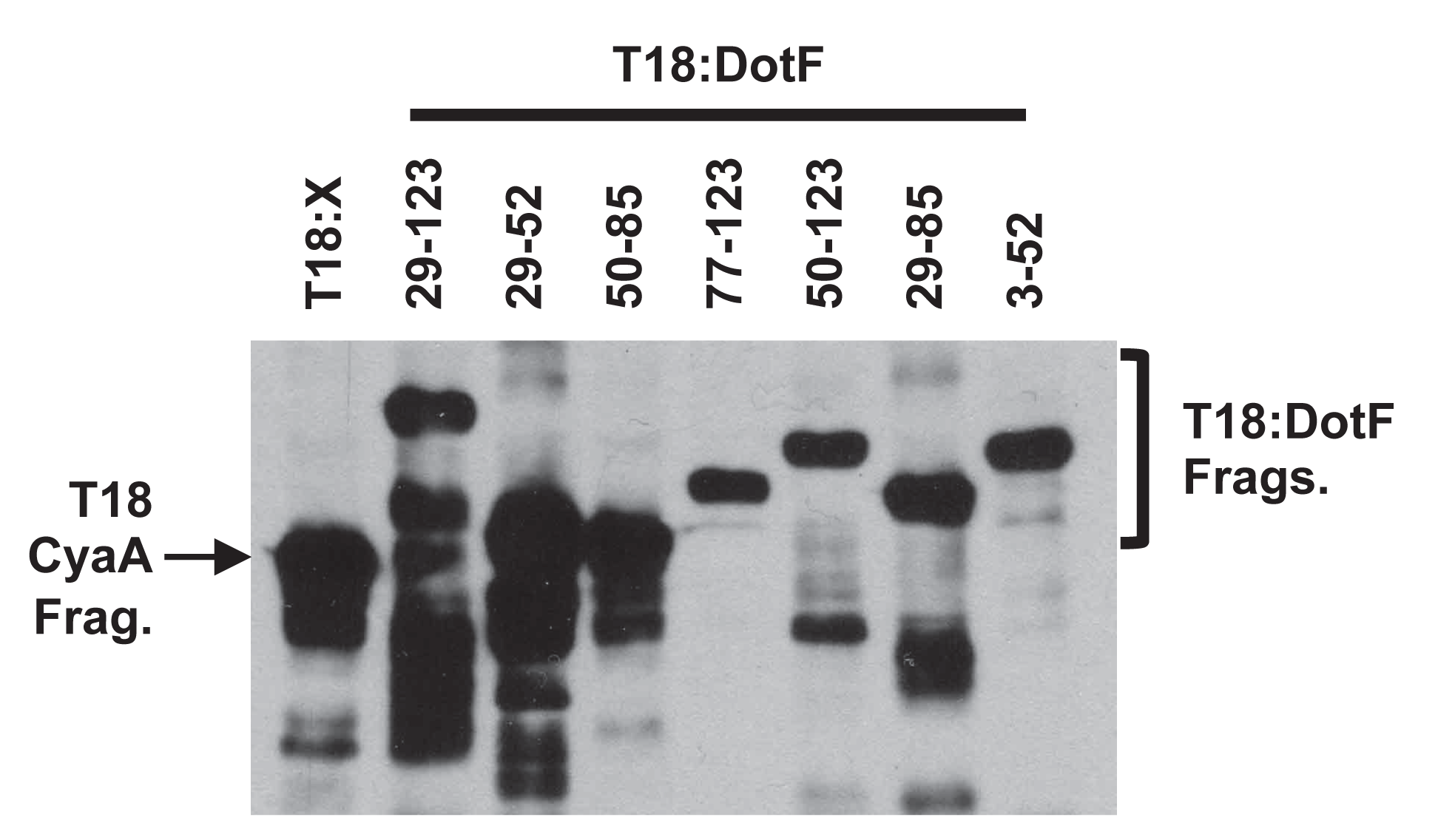

Supplement: Figure S1 — The T18:DotF two-hybrid constructs produce equivalent amounts of protein. The T18:DotF protein levels were assessed by western blot with a CyaA-specific antibody. The T18 CyaA fragment expressed from the vector is indicated by an arrow. The T18:DotF fragments are designated by a bracket and their amino acid range is indicated above the western blot. (TIF) [file pone.0065529.s001.tif]
