# Supplementary material for: Reassessing the Role of DotF in the Legionella pneumophila Type IV Secretion System
Source: PLoS One. 2013 Jun 7;8(6):e65529. doi: 10.1371/journal.pone.0065529 (PMC3676331; doi:10.1371/journal.pone.0065529)
Supplement: Table S1 — Dot/Icm T4SS substrates tested for interaction with DotF(29–123). (PDF) [file pone.0065529.s002.pdf]

Table S1. Dot/Icm T4SS substrates tested for interaction with the DotF(29-123) fragment

Substrate	Lpg#	DotF interaction on MacConkey agar	Miller Units
LnaB	2527	+	3048 +/- 353
RalF	1950	+	3706 +/- 227
SdeA	2157	+	1375 +/- 55
SidF	2584	+	2403 +/- 72
SidG	1355	+	2256 +/- 81
SidJ	2155	+	259 +/- 11
LegG2	0276	-	ND
LidA	0940	-	ND
SdcA	2510	-	ND
SdhA	0376	-	ND
SidD	2465	-	ND
SidM	2464	-	ND
VipA	0390	-	ND
WipA	2718	-	ND
YlfA	2298	-	ND

+ = red pigment/positive interacion
- = no pigment/negative interaction
ND = not determined
