# Supplementary material for: Reassessing the Role of DotF in the Legionella pneumophila Type IV Secretion System
Source: PLoS One. 2013 Jun 7;8(6):e65529. doi: 10.1371/journal.pone.0065529 (PMC3676331; doi:10.1371/journal.pone.0065529)
Supplement: Table S2 — Strains, plasmids and primers employed in this study. (PDF) [file pone.0065529.s003.pdf]

Table S2. Relevant strains, plasmids and primers employed in this study

Strain, Plasmid, Primer	Relevant Properties	Reference or Source
Legionella		
Lp02	Philadelphia-1 *rspL hsdR thyA*	Berger and Isberg, 1993
Lp03	Lp02 *dotA*	Berger and Isberg, 1993
JV1139	Lp02 + pJB908	Bardill et al, 2005
JV1141	Lp03 + pJB908	Bardill et al, 2005
JV2064	Lp02 Δ*dotA*	Vincent et al, 2006
JV3579	Lp02 Δ*dotF*	Vincent et al, 2006
JV3901	Lp02 + CyaA:RalF (pJB2590)	Bardill et al, 2005
JV5710	Lp02 + CyaA:SidG (pJB4520)	This study
JV5961	Lp02 Δ*dotA* + CyaA:SidG (pJB4520)	This study
JV7894	Lp02 Δ*dotF* + CyaA:RalF (pJB2590)	This study
JV8581	Lp02 Δ*dotF* + pJB908	This study
JV8582	Lp02 Δ*dotF* + *dotF* (pJB2121)	This study
JV8700	Lp02 Δ*dotA* + CyaA:RalF (pJB2590)	This study
JV8801	Lp02 + CyaA:RalF(ΔSS) (pJB6489)	This study
JV8802	Lp02 + CyaA:SidG(ΔSS) (pJB6490)	This study
JV8803	Lp02 Δ*dotA* + CyaA:RalF(ΔSS) (pJB6489)	This study
JV8804	Lp02 Δ*dotA* + CyaA:SidG(ΔSS) (pJB6490)	This study
JV8807	Lp02 Δ*dotF* + CyaA:SidG (pJB4520)	This study
E. coli		
BL21	F^- *dcm ompT hsdS*(r_{B-} m_{B-}) gal	Novagen
BTH101	F^-, *cya-99*, *araD139*, *glE15*, *galK16*, *rpsL1*, *hsdR2*, *mcrA1*, *mcrB1*	Karimova et al, 1998
Plasmid		
pKB5	RSF1010 cloning vector, *thyA*$^+$, *bla*, *mob*	Berger and Isberg, 1993
pKT25 (pJB2774)	T25:X p15A *ori*, Kan^R cloning vector	Karimova et al, 1998
pUT18C (pJB2777)	T18:X ColE1 *ori*, Amp^R cloning vector	Karimova et al, 1998
pJB908	pKB5 Δ*oriT*	Sexton et al, 2006
pJB2121	*dotF* complementing clone	This study
pJB2581	CyaA:X fusion vector RSF1010 Amp^R Cm^R Δ*oriT*	Bardill et al, 2005
pJB2590	CyaA:RalF	Sutherland et al, 2012
pJB3322	T25:X p15A *ori*, KanR cloning vector	This study
pJB3540	T18:X ColE1 *ori*, Amp^R cloning vector	This study
pJB3667	T25:DotF(29-123)	This study
pJB3674	T18:DotF(29-123)	This study
pJB4520	CyaA:SidG	This study
pJB6485	T25:SidG(ΔSS)	This study
pJB6489	CyaA:RalF(ΔSS)	This study
pJB6490	CyaA:SidG(ΔSS)	This study
pJB6491	T25:RalF(ΔSS)	This study
pJB6600	T25:RalF(ΔSS)	This study
pJB6601	T25:SdeA	This study
pJB6607	T25:SidJ	This study
pJB6608	T25:SidF	This study
pJB6611	T25:LnaB	This study
pJB6612	T18:RalF(ΔSS)	This study
pJB6613	T18:SdeA	This study
pJB6619	T18:SidJ	This study
pJB6620	T18:SidF	This study
pJB6623	T18:LnaB	This study
pJB6636	T25:SidG	This study
pJB6638	T18:SidG	This study
pJB6675	T18:DotF(29-52)	This study
pJB6676	T18:DotF(3-52)	This study
pJB6677	T18:DotF(29-85)	This study
pJB6678	T18:DotF(50-123)	This study
pJB6697	T18:DotF(77-123)	This study

pJB6814	T18:DotF(50-85)	This study

Primers

JVP0541	CCCGGATCCAAACCTTTTGAGTTGAATTTCG
JVP0543	CCCGGATCCCATCCAGAAATTGAAAAAGCCC
JVP0620	CCCTCTAGATGGCGGATAATGATGGCAG
JVP0621	CCCGTCGACGGATCAGCCATATACGCC
JVP0856	GGGGGATCCCCTAAGTATGTCGAAGGGGTAG
JVP0857	GGGGTCGACACTTCATGCGATTTGGGTTAAGG
JVP1106	CCCGGATCCTTTGGTTTCATAAAGAAAGTACTTG
JVP1107	CCCGTCGACCAAACGTTTATCAGTAGTACGTTCG
JVP1225	CTTGGATCCCCCGGGCTCGAGCTGCAGGCATGCGGCCGCGGTAC
JVP1226	ACGTGAACCTAGGGGGCCCGAGCTCGACGTCCGTACGCCGGCGC
JVP1251	CCCGGATCCGCAGAGCACGATCAAAATAATGATG
JVP1264	TCGGATCCCCCGGGCTCGAGCTGCAGGCATGCGGCCGCGGTAC
JVP1265	CGCGGCCGCATGCCTGCAGCTCGAGCCCGGGGGATCCGATGCA
JVP1583	GCGGGATCCGATCTCGATTCAGAAGCCTCG
JVP1584	GCGGTCGACTCAATCGGGGTCATCTTCAATAATAGTCG
JVP1679	GCAGTCGACTCCCGTTCGAAAGATGAAGTTTTAGAAGC
JVP1680	GCACTGCAGTTATATGCGCATCGTACTTTCAGTTCC
JVP2294	GCAGGATCCCCACGAATCACTGAAAATATAGAAACC
JVP2295	GCAGTCGACCCCTTAGAAGTTTACTGGCGTGG
JVP2325	GCAGGATCCTCATACCAAAAAATAGAACTCAGTATTTC
JVP2326	GCAGTCGACGCAAGAGATCTATTGTAAACGAGC
JVP2412	CCAGTCGACGAATGATTTATTCTTAGTCTAATTGG
JVP2556	CCCGTCGACTTACTCCTTAAGTGCCAGGTTTCTCTC
JVP2559	GCGGTCGACTCAATTTCGCTTAATATCTTTTTTCTTTGTTAATCCC
JVP2560	GCGGTCGACTCACTTTGAGGTTACTTGGCTGGATTTGTC
JVP2561	GCGGGATCCAAGCGAAATGCTCTTATTGCTATTGG
JVP2576	GCAGGATCCGACAAATCCAGCCAAGTAACCTC
JVP2706	GCACTGCAGTTAAGTTAATTCCGTTTTATCAGTAGATGTAACTTCTTCC
JVP2717	GCAGCGGCCGCTTAAGTTAATTCCGTTTTATCAGTAGATGTAACTTCTTCC
