# Supplementary material for: Reassessing the Role of DotF in the Legionella pneumophila Type IV Secretion System
Source: PLoS One. 2013 Jun 7;8(6):e65529. doi: 10.1371/journal.pone.0065529 (PMC3676331; doi:10.1371/journal.pone.0065529)
Supplement: Table S3 — Construction of plasmids employed in this study. (PDF) [file pone.0065529.s004.pdf]

Table S3. Construction of plasmids employed in this study

Plasmid	Relevant Properties	Vector[a]	Enzymes used to digest vector	Primers[b]	Enzymes used to digest insert
pJB2121[c]	*dotF* complementing clone	pJB908	XbaI/SalI	JVP620/JVP621	XbaI/SalI
pJB3322[d]	T25:X cloning vector	pJB2774	PstI/KpnI	JVP1225/JVP1226	PstI/KpnI
pJB3540[e]	T18:X cloning vector	pJB2777	PstI/KpnI	JVP1264/JVP1265	PstI/KpnI
pJB3667	T25:DotF(29-123)	pJB3322	BamHI/SalI	JVP1583/JVP1584	BamHI/XhoI
pJB3674	T18:DotF(29-123)	pJB3540	BamHI/SalI	JVP1583/JVP1584	BamHI/XhoI
pJB4520	CyaA:SidG	pJB2581	SalI/PstI	JVP1679/JVP1680	SalI/PstI
pJB6485	T25:SidG(ΔSS)	pJB3322	SalI/NotI	JVP1679/JVP2717	XhoI/NotI
pJB6489	CyaA:RalF(ΔSS)	pJB2581	BamHI/SalI	JVP543/JVP2556	BamHI/SalI
pJB6490	CyaA:SidG(ΔSS)	pJB2581	SalI/PstI	JVP1679/JVP2706	SalI/PstI
pJB6491	T25:RalF(ΔSS)	pJB3322	BamHI/SalI	JVP541/JVP2556	BamHI/XhoI
pJB6600	T25:RalF	pJB3322	BamHI/SalI	JVP541/JVP2412	BamHI/XhoI
pJB6601	T25:SdeA	pJB3322	BamHI/SalI	JVP856/JVP857	BamHI/XhoI
pJB6607	T25:SidJ	pJB3322	BamHI/SalI	JVP1106/JVP1107	BamHI/XhoI
pJB6608	T25:SidF	pJB3322	BamHI/SalI	JVP2294/JVP2295	BamHI/XhoI
pJB6611	T25:LnaB	pJB3322	BamHI/SalI	JVP2325/JVP2326	BamHI/XhoI
pJB6612	T18:RalF	pJB3540	BamHI/SalI	JVP541/JVP2412	BamHI/XhoI
pJB6613	T18:SdeA	pJB3540	BamHI/SalI	JVP856/JVP857	BamHI/XhoI
pJB6619	T18:SidJ	pJB3540	BamHI/SalI	JVP1106/JVP1107	BamHI/XhoI
pJB6620	T18:SidF	pJB3540	BamHI/SalI	JVP2294/JVP2295	BamHI/XhoI
pJB6623	T18:LnaB	pJB3540	BamHI/SalI	JVP2325/JVP2326	BamHI/XhoI
pJB6636	T25:SidG	pJB3322	SalI/PstI	JVP1679/JVP1680	XhoI/PstI
pJB6638	T18:SidG	pJB3540	SalI/PstI	JVP1679/JVP1680	XhoI/PstI
pJB6675	T18:DotF(29-52)	pJB3540	BamHI/SalI	JVP1583/JVP2559	BamHI/XhoI
pJB6676	T18:DotF(3-52)	pJB3540	BamHI/SalI	JVP1251/JVP2559	BamHI/XhoI
pJB6677	T18:DotF(29-85)	pJB3540	BamHI/SalI	JVP1583/JVP2560	BamHI/XhoI
pJB6678	T18:DotF(50-123)	pJB3540	BamHI/SalI	JVP1584/JVP2561	BamHI/XhoI
pJB6697	T18:DotF(77-123)	pJB3540	BamHI/SalI	JVP2576/JVP1584	BamHI/XhoI
pJB6814	T18:DotF(50-85)	pJB3540	BamHI/SalI	JVP2561/JVP2560	BamHI/XhoI

[a]See Table S2 for pJB908, pJB2581, pJB2774, pJB2777 references

[b]Primer sequences are provided in Table S2

[c]Example of cloning procedure (pJB2121):

dotF was amplified using primer JVP620/JVP621. The PCR product was digested with XbaI/SalI and ligated into pJB908 digested with XbaI/SalI

[d]Detailed description of pJB3322 construction:

pKT25 (pJB2774) was modified to include a new multicloning site (MCS). JVP1225/JVP1226 were annealed and ligated into pJB2774 digested with PstI/KpnI. The new MCS consists of the following restriction sites: BamHI, SmaI, XhoI, PstI, SphI, NotI, KpnI.

[e]Detailed description of pJB3540 construction:

pUT18C (pJB2777) was modified to include a new MCS. JVP1264/JVP1265 were annealed and ligated into pJB2777 digested with PstI/KpnI. The new MCS consists of the following restriction sites: BamHI, SmaI, XhoI, PstI, SphI, NotI, KpnI, SacI, EcoRI, ClaI.
